# Use of patients’ unsolicited correspondence to a family doctor to describe and understand valued components of a doctor-patient relationship: A Hermeneutics approach

**DOI:** 10.1186/s12875-019-1024-6

**Published:** 2019-10-17

**Authors:** Mark J. Yaffe, Richard B. Hovey, Charo Rodriguez

**Affiliations:** 1 Department of Family Medicine, McGill University, 5858 Côte-des-Neiges Road, suite 300, Montreal, Quebec H3S 1Z1 Canada; 2 St. Mary’s Hospital Center, Family Medicine Centre, 3830 Lacombe Avenue, Montreal, Quebec H3T1M5 Canada; 3 Integrated University Centre for Health and Social Services of West Island of Montreal, Family Medicine Centre, 3830 Lacombe Avenue, Montreal, Quebec H3T1M5 Canada; 4 Division of Oral Health and Society, Faculty of Dentistry, McGill University, 2001 McGill College, suite 500, Montreal, QC H3A1G1 Canada

**Keywords:** Doctor-patient relationship, Hermeneutic enquiry, Patient correspondence, Patient-centered care, Hippocratic medicine, Asklepian healing

## Abstract

**Background:**

Communication and behavior within doctor - patient encounters have been examined using varied techniques; however the nature of unsolicited writings from patients to their family doctors has rarely been reported. This paper therefore aimed to explore the content of, and motivation for, such correspondence.

**Methods:**

One hundred and seven writings to one family physician about care provided during a four decade period were considered. Univariate analyses were used to identify features of patients or family members who wrote personalized notes to the doctor, when, and in what fashion. A hermeneutic approach helped look at the content of the notes, the specific words or sentiments used to describe encounters or care received, and possible motivations for writing. Iterative review of words or phrases generated themes which summarized appreciated physician or relational attributes, as well as motivations for writing.

**Results:**

Notes were mostly handwritten, predominantly by women, and frequently coinciding with holidays and life span events. Appreciated doctor characteristics and behaviors were (1) quality care; and physician (2) competence; (3) physical presence; (4) positive personal traits; (5) provision of emotional support; and (6) spiritual impact. Motivations for writing were grouped as desire to (1) express appreciation for an established relationship; (2) acknowledge value / benefit experienced from continuity of care; (3) seek catharsis, emotional relief or closure; (4) reflect on termination of care; (5) validate care that incorporates both Hippocratic tradition and Asklepian healing; and (6) share personal reflection, experience, or impact.

**Conclusions:**

Unsolicited writings provide personalized links from patients to physicians, expressing thoughts perhaps difficult to share face to face. They offer potential as teaching tools about the content of doctor-patient relationships; for example, the writers studied expressed appreciation for quality continuity care that was competent, considerate, and supportive of emotional and spiritual needs.

## Background

Positive doctor-patient relationships have been linked to improvements in patients’ health [1], and lower symptom burden, rates of referral [2] and diagnostic costs [3]. In a family practice setting where continuity of care is encouraged across a bio-psycho-social-ethical-spiritual spectrum, how are such relationships perceived or experienced by patients and /or their family members? To answer this question quantitative methodologies have used as many as 19 different instruments to study these relationships [4], while qualitative studies have examined illustrative case histories, physician or patient narratives [5, 6], direct observation of doctor – patient interactions (audio or video recorded for later analysis), and semi-structured focus group discussions [7]. Art, literature, movies, and theatre have, to varying extents, portrayed vignettes of different types of clinical care [8–10].

Patients and /or their family members are known to occasionally write to their doctors, suggesting another possible window onto doctor-patient encounters. However, in association with this paper, a literature review by one of us (MJY) designed to identify in-depth articles examining the content of such letter writing to family doctors, found only one publication. Specifically, Merenstein and Merenstein [11] described correspondence from patients written to commemorate the retirement of one of them. Composed at a time common to all the writers, it documents patients’ reflections and appreciations of past care.

Perception of personal experiences may change over time, however, influenced by the world around us, and by prejudices, traditions, expectations, and changing patterns of professional behavior. As well, changing values, emotional investment, and vocabulary usage may also be temporal. We consequently wondered what could be learned about doctor-patient relationships from an examination of correspondence that was written over a broader expanse of time or was more proximal to actual events or experiences. We decided, therefore, to examine the nature and content of a collection of patients’ and family members’ unsolicited cards and letters sent to one family doctor, written at will over a period of four decades. The research questions that guided our work were: (1) Amongst patients or family members, who wrote to the family doctor, when, and in what fashion? (2) What were the apparent motivations or spirits underlying the writing of the notes? (3) What did their writings suggest they valued in the relationship with, or care from, the doctor?

## Methods

### Hermeneutic tradition

The approach taken to understanding the meaning associated with written correspondence to the doctor was Hermeneutics, a philosophical tradition founded in the ontological understanding that the world is interpretable, and that we as humans are always in the process of interpreting that which surrounds us [12]. The word hermeneutics is derived from the Greek god Hermes, who was tasked with exchanging messages between the gods and humans [13]. The challenge for Hermes was to bridge differences in the ways of knowing and thinking that existed between the gods and humans. He would sometimes therefore not deliver his messages in a straightforward, clear or concise way, but rather in a form that provoked reflection and deeper thinking.

By the twentieth century Hermeneutics was seen as the art of interpretation which applies to all forms of human understanding [12]. The hermeneutic theory of experience is explained along with the notion of understanding, emphasizing the way in which experience and understanding are always connected, and how practical knowledge is acquired by direct evidence from those who have had an experience, unique or otherwise. That is, they are interconnected insofar as they require active participation, and in this context, experience takes on both a cognitive and tangible meaning, which is neither simply epistemological nor intellectual, but rather practical.

Empirical knowledge gained from professional practice is basically that which everyone accumulates as they become a doctor, dentist, nurse or any kind of health practitioner. Not only in the professional domain but also in everyone’s private and personal life, the experience that people develop from an encounter with themselves and other human beings continually grows and evolves, as we become experienced [14]. Within the “hermeneutic experience” the reading and interpretation of letters from patients and families may provoke critical self-reflection which may disrupt or challenge professional and personal ways of understanding, which may determine capacity to engage with others or with written text [13].

### Source of study material

During residency training over four decades ago one of us (MJY) received a couple of letters expressing appreciation for care received. They were encouraging and motivating, leading to their retention, along with comparable correspondence received over 40 years of medical practice in an urban-based teaching Family Medicine Center, within a multi-ethnic secondary care community hospital affiliated with McGill University in Montreal. This resulted in a collection of 140 pieces of correspondence, written in the preferred language of the patient or family (English or French). Of the 140, 33 contained no personalized writings; rather they were expressions of appreciation of care as they appeared either in notices of charitable donations, or in newspaper obituary announcements. Since the goal of the study was to examine writer choice of words and sentiments about the care provided by the physician, the 33 were excluded from analysis.

### Identification of writers

The initial reading of the notes focused on identifying characteristics of the writers (gender, estimated age, patient or family member). This was performed by MJY uniquely, since the copies of the notes that were used by the other members of our team had demographic identifiers and names blacked out for confidentiality reasons. We then collectively made note of the time of year or the situation around which each correspondence was written; the different forms the correspondence took; and particular words used to describe attributes of the physician or the care provided.

### Application of hermeneutic interpretation

A hermeneutic approach was then undertaken for analysis of words in each correspondence that might give clues as to what prompted the writings and the particular feelings underlying them. Since Hermeneutics assumes that any written note has the potential to evoke memories, mental images of an event, disclosures, new insights or an expanded insight, a formalized oral reading of the correspondence was made by MJY, with prompts, enquiries, or requests for clarifications by RBH or CR. This process attempted to overcome possible change in word meaning over time, in order to present an initial semantic interpretation of patients’ experiences of family physician care. These discussions were digitally recorded and then transcribed to retain a written record of memories and ideas that had been elicited. Based on this stimulated recall, the interpreted motivations or spirit for writing were grouped along common themes, recognizing that with complex content, some overlap in groupings was possible.

## Results

The 107 notes fit into three physical formats: 58 handwritten on blank paper; 32 handwritten on an array of various commercial cards; and 17 typed on blank paper. Three quarters of the notes were written by women (spouse, widow, or daughter of a patient). The timing of the note writing appeared to be predominantly around four life course events or issues: (1) secular or religious holidays; (2) in conjunction with a birth, illness, recovery, death, or anniversary of a death; (3) a geographical move necessitating a leave from the practice; and (4) a need to reach out for uniquely personal reasons.

Our review of the notes for specific words used by the writers to depict the doctor-patient encounters and the doctor’s personal attributes, identified 186 such descriptors, an average of 1.7 per note. A grouping of the 186 according to comparable meanings reduced this to 44 discrete words or phrases, as summarized in Table 1.

**Table 1:** Frequency of cited physician attributes and related themes

Physician Attributes	Frequency	Themes
Good / Excellent care	18	Competence
Caring	12	Emotional support
Kind / Kindness	11	Personal trait
Shows patience	10	Personal trait
Understanding	9	Emotional support
Support, Moral support	9	Emotional support
Concerned	8	Emotional support
Being there for me us	8	Physical presence
Expertise	8	Competence
Attentive	8	Quality of care
My / Our friend	7	Spiritual impact
Compassionate	6	Emotional support
Humane	6	Personal trait
Listens / takes time to listen	6	Physical presence
Sympathetic	5	Emotional support
Shows professionalism	5	Competence
Devoted	4	Quality of care
Gives extra time	4	Physical presence
Helps when needed	4	Physical presence
Thoughtful	4	Competence
Gives guidance	4	Spiritual impact
Available	3	Physical presence
Empathic	2	Emotional support
Shows respect	2	Emotional support
Never gives up on me	2	Physical presence
Skillful	2	Competence
Wisdom / depth of thought	2	Competence
Genuine	2	Personal trait
Engenders faith	1	Spiritual impact
Engenders hope	1	Spiritual impact
You are my doctor	1	Spiritual impact
Makes a difference	1	Spiritual impact
Part of my world	1	Spiritual impact
Shows judgement	1	Competence
Medically wise	1	Competence
Helped with heart and soul	1	Personal trait
Nice	1	Personal trait
Someone I can talk to	1	Personal trait
Shows sensitivity	1	Personal trait
Comprehensive	1	Quality of care
Dedicated	1	Quality of care
Explains	1	Quality of care
Gives prompt response	1	Quality of care
Shows constancy	1	Physical presence

The 44 further lent themselves to six summative themes that described appreciated or valued physician qualities or relationship attributes: (1) quality of care; and physician (2) competence; (3) physical presence: (4) positive personal traits; (5) provision of emotional support; and (6) spiritual impact.

The interpretive hermeneutic approach of examining the content of each note, followed by stimulated review of events recalled about the patient and /or family, permitted the identification of specific phrases that provided suggestions as to six contexts, motivations or spirits underlying the writing of the notes: (1) expression of appreciation for an established relationship; (2) acknowledging value / benefit of continuity of care; (3) seeking catharsis and /or emotional relief or closure; (4) reflecting on termination of care; (5) validating the process and content of care that extended beyond the Hippocratic tradition into that of Asklepian healing [18]; and (6) sharing personal reflection, experience, or impact. Selected quotations from patients and / or family members cited below illustrate these motivations:

### Express appreciation for established relationships

- ❖ A married mother of 3 young children wrote to express thanks for help during the previous year: *“There is nothing we could say to show you how much we appreciate you as a friend and Doctor.”*
- ❖ A daughter, writing to express thanks for the care her mother received over some 30 years: *“She always had strong memories of you and would relate the same to us. You were a big part of her life, and with that, ours as well.”*
- ❖ A 55 year old woman recovering from a lengthy illness reflected on friendship: “*I have been asked many times about what a friend is and now I know……it is someone who is there for you, you, no matter what, and you are my friend.”*
- ❖ A woman in need of a doctor for an acute gynecological problem saw her husband’s family doctor and observed: *“You take the time to listen, and you explain thoroughly and patiently. You bridge the gap between doctor and patient and you give medicine its much needed human touch.”*
- ❖ Following a visit to his 87 year old mother, an out of town son wrote: *“We all feel blessed that our mother is in your care and recognize her deep sense of gratitude to you for the way you respect and treat her.”*
- ❖ A Season’s Greeting card from a 73 year old woman reflected on *“over three decades of our doctor-patient relationship”: “Thank you for being ‘my’ physician for such a long time that is going back to my parents and good husband.”*

### Acknowledging value / benefit of continuity of care

- ❖ A woman in her early 70’s wrote about the care she and her husband had received: “*I am the envy of my friends in having such an attentive and dedicated family doctor……I tell them to choose doctors twenty to thirty years younger so that they will be around when needed.”*
- ❖ An adult daughter, reflecting on her parents, was appreciative of the “*many years of care you were the family doctor---with guidance, feedback, advice, and caring ---invaluable to their health and our peace of mind”.*
- ❖ A woman in her late twenties who had been struggling with both career and relationship issues wrote: *“Thank you for your kind, thoughtful and continuous ministerings. It was a tremendous help to be able to talk with you and get the guidance and reassurance I needed.”*
- ❖ An out-of-town daughter wrote about her parents’ illnesses: *“I know how much Mum appreciated your care and support during my father’s decline. Now she again relies so much on your expertise, presence, and care in her current situation. No one can really say what the next few weeks will bring but she will derive comfort ( and so will we) from knowing that you have been part of her decision-making process and that you will be there whether or not she calls on you.”*

### Seeking catharsis and /or emotional relief or closure

- ❖ A woman undergoing serious marital problems wrote: *“Thank you for seeing me last Tuesday. Talking to someone that cares and understands made me feel good. I am a very private person and I don’t get to talk about private matters to many people.”*
- ❖ Grieving her 93 year old mother-in-law, a daughter-in-law appeared to find solace in the following reflection: *“We will always remember your caring attentiveness to……, with appreciation and deep respect”.*
- ❖ A condolence note from the doctor to the family of a man who had a sudden death evoked this family response: *“Your described impression of…..captured some of his key attributes and this touched us deeply.”*
- ❖ A daughter, writing following the death of her father, wrote: *“it was touching to have you come to the visitation”.*
- ❖ Following the funeral of her elderly husband his widow wrote: *“Thank you for coming to share in our pain….He always had great faith in you as his physician and we are grateful for the concern and compassion you showed us during those difficult last days in the hospital.”*
- ❖ On the second anniversary of her husband’s death a widow wrote: *“I have been meaning to write……it is amazing how fast the time goes…….I think about all the times you were giving of yourself as a true and dedicated physician always at the ready, no matter what the medical problem was involved, it was solved by your expertise and care.”*

### Reflecting on termination of care

- ❖ A retired couple, hesitant to move 75 km to a new community and away from their doctor, wrote: *“on your advice we have finally cut the cord from your 25 years of excellent care and have managed at last to find a family doctor in …….”*
- ❖ Also retiring and moving away a woman in her mid-sixties observed: *“...having come from a medical family myself, I very much appreciated how comprehensive and in-depth and reassuring I found your check-ups to be.”*
- ❖ A couple in their late 60’s, unnerved by city traffic, sought help to find a doctor closer to their home. *“Thanks to you, to your attentive listening, to your follow-up, to your decisions and treatment orientations which we immensely appreciated, we are continuing to enjoy relatively good health.”* (translated from French)
- ❖ A woman in her early 30’s, in the practice since her teens, wrote on getting married and moving away: “*thank you for all the years you were my doctor. I appreciate all the help, advice, and the kindness. You are one of the few physicians I have met who still knows the value of sympathetic and gentle medical attention.”*

### Validating Asklepian healing

- ❖ On finishing chemotherapy a woman in early 70’s wrote: *“When I took sick last summer, I had the peace of mind knowing that you were there to see that I got the best of care. I think this is what has enabled me to get through it all with success.*
- ❖ A woman, distraught over her young grand-daughter’s very serious illness wrote: *“..thank you again for the time you spent with me during ‘my time of woe’ ....it was comforting to know you took the time to reassure me.”*
- ❖ Following an intense, emotionally-laden interaction with the family of a hospitalized patient, the hospital chaplain wrote to the physician: *In your role as healer of the sick you are a true manifestation of God’s love.”*
- ❖ Reflecting on her mother’s struggle with cancer, her only child wrote: *“……without his {*the doctor’s} *devoted and humane care—which went far beyond the call of duty—she would not have been able to have the time and the sense of well-being that she could.”*
- ❖ A 42 year old mother of a chronically ill child wrote: *“…I never expected to descend upon your office in a three hour crisis. But you didn’t make me feel embarrassed or hysterical---you calmed me down with a combination of logic and empathy. You always seem to have a simple effective solution, and you make me feel connected (rather than disconnected) to the world.”*
- ❖ A 75 year old woman, reflecting on the health deterioration of her husband, wrote: *“Thank you for letting me personally experience your values in practicing FAMILY MEDICINE in the noblest sense of the concept……..above all, thank you for trying to give me a ray of hope within the restraints of reality, in the midst of the darkest days.”*

### Need to share personal reflection, experience, or impact

- ❖ Inside a Season’s Greetings card the sender added: *“With good medical care, being over ninety is not so bad after all!”*
- ❖ Following the death of his 92 year old father, a son expressed appreciation for *“your professionalism, caring and ability to care for a senior who, at times, let’s face it, could be less than easy to work with!”*
- ❖ A woman in her late 50’s, writing on behalf of herself and family, wrote: *“We are not people of many words, but we thank you for your kindness, understanding, high professionalism, and so much humanity…….you are not only our family physician but truly a good friend who helped us to overcome moments of real difficulty, helping us to have answers to so many questions.”*
- ❖ A woman in her early 30’s, noted for turbulent teen-age and early adult years, shared her feelings: *“Thanks for never giving up on me during my dark years and being a constant helper – many times by saying ‘NO’ to me.”*
- ❖ A physician, expressing thanks for the care of his deceased mother, observed: *“She had much esteem and respect for you as a person and as a physician. Coming from her, this was no small compliment.”*
- ❖ A usually self-sufficient problem-solver in her 70’s wrote after a crisis: *I am very fortunate that I have the privilege of being your patient. Even with a schedule that can only be defined as “hectic” you always find the time for situations that require your attention.”*
- ❖ Mr. G., lived in a government-financed foster home, dealing with alcoholism, hepatic encephalopathy, slurred speech, urinary incontinence and low self-esteem. The family doctor visited him every 3 months for evaluation, encouragement and addressing his solitude. Mr. G. inscribed in a starkly simple white commercial card the words: *“It’s great to have good friends like you who think of such kind things to do.”*
- ❖ A social worker wrote the doctor to share a conversation she had with an elderly patient they jointly cared for: “*He does not like many people… but he said he likes you and he called you a ‘Tzadik’ (a Hebrew word in the Jewish tradition to mean a ‘Righteous Person’).”*

## Discussion

This paper set out to explore the content and meaning of forty years of unsolicited correspondence sent by patients and /or family members to one family doctor. It is not intended as a commentary on a specific physician; rather, an enduring incentive for retention of this material over such a lengthy time was the hope that its later examination as a collection might contribute to an understanding of what patients value in the clinical encounter.

The nature of Hermeneutics is to interpret human experience, whether through text, actions, stories or other forms of expressions, and thus provide insight into human expressions [15]. The philosophical hermeneutical approach used in this research was an interpretative one that looked at the meaning and experience of the doctor-patient encounter as interpreted from four decades of personal notes written by patients and /or family members to their family physician. While the words written on these notes denote a sense of gratitude and appreciation, their deeper meaning lies within the context and experience within each patient-physician engagement, which appears to have been the motivation for these writings.

Our use of the hermeneutic interpretative enquiry would thus seem to be a strength of this work. The correspondence chronicles challenging events that occurred in the lives of people, who while differing in backgrounds, ages, and concerns, may be seen as a definable community in that they sought help and care from a particular doctor. This may have been the result of chance, geographical factors, recommendation or referral by others, or perhaps preference for a particular doctor personality, practice style, or professional interest.

The notes were mainly written by women, likely the result of their predominant roles as caregivers to ill relatives, as well as family nurturers, and in some cases, having simply outlived their male spouses and relatives. These writings seem to bear witness to such roles and their related experiences. For others they may have offered the writers an opportunity to show their own humanities and uniqueness.

This individuality may be seen, for example, in the choice of when to write. Some corresponded specifically around the time of a secular or religious holiday; some showed additional thoughtfulness by doing this at a time they believed was meaningful to, or celebrated by, their doctor. The personality of the writer might also be surmised by how the notes were written, with just over half containing handwritten thoughts that completely filled a page or more. An additional third were commercial cards with printed messages, suggesting perhaps special effort had been made to purchase ones with specific personal meaning; interestingly, some of these also contained short hand-written comments to add to the personalization of these notes. Finally, a small number came typed, suggesting possible formality of the writers; however since the wording was not necessarily formal, the typing might have been a considerate means of sparing the reader a handwritten script that was difficult to read.

The correspondence from patients and families is noteworthy for the commonalities in words and themes that describe what was valued in the family physician-patient / family encounters. Iterative attempts were made to combine them within common groupings, and some disagreement may arise about any specific allotment since there was indeed, some potential for overlap within groups. We do not, however, believe that this affects the overall interpretations in this paper.

What patients and families appeared to have valued was quality of care, and physician characteristics such as competence, physical presence, positive personal traits, emotional support, and spiritual impact. These descriptors encompassed care that was knowledgeable bio-medically, and experienced as available, authentic, reality-based, supportive, professional, compassionate, offering of hope, and sensitive to suffering. As well, six distinct contexts or motivations for writing to doctors have stood out in this work: (1) opportunity for expression of appreciation for an established relationship; (2) acknowledging value / benefit of continuity of care; (3) a means of seeking catharsis, emotional release, and closure; (4) a chance to reflect on termination of care; (5) a recognition of care that extended into the Asklepian healing tradition [16]; and (6) opportunity to share personal reflection, experience, or impact.

A limitation in this project may be that no negative comments or remarks have been cited; indeed, none were actually found in the 107 notes. Since it is reasonable to conclude that not all people are satisfied with care all of the time, what of their letters? Stimulated by relevant questioning about this from the other authors, MJY could not recall having discarded derogatory letters, although that remains a possibility. Four other hypotheses are suggested: Firstly, a feeling of discontent might not have been great enough to prompt writing a letter. Secondly, an unhappy patient might have addressed concerns, directly, face to face, with the doctor during an office visit. Thirdly, an unsatisfied patient might have left the practice without indication or notification. Finally, with the contemporary emphasis on quality assurance, patient representatives and ombudsmen, it is possible that some patient and /or family dissatisfaction got diffused at that level.

In summary, patients and /or their families in this study have used unsolicited correspondence to a family doctor to identify what they value in the doctor and in the process of care. It is reassuring that some of the concepts we identified appear to be generalizable in that they were also reported by Merenstein and Merenstein [11]. Their work, however, was associated with a specific point in time, a physician retirement, when nostalgia might have influenced the content of the writings. Our findings are likely broader in that they are more numerous in number, the product of forty years of writing, and composed by both past and current patients. We have, as well, been able to take our analysis further by exploration of the contexts and motivations under which people to their doctors.

It is likely that letter writing may have provided personal psychological benefits for the writers. What might one surmise would be the impact of patient/family letters on physicians? The letters may serve as feedback about how the care they are giving is perceived. If positive, they may serve as validation, promoting positive mental health, possible personal resiliency and longevity in practice. On the other hand, even if generally laudatory, a doctor must be alert to the heterogeneity of patients in the practice and be cognizant that their needs and reactions may differ from those expressed in the letters. If the content is negative the challenge would be for the physician to maintain positive countertransference and engage the patient to explore where problems might exist.

## Conclusions

Letters and notes from patients and family may be a potential teaching tool since they provide trainees with a personalized, “real-life” attestation of what people seeking health care want.

The notes in this study suggest patients appreciate medical care in which Hippocratic and Asklepian traditions overlap and complement each other [16]. The findings suggest that health care models based on walk-in style care, rapid visits, or low access to a constant primary care physician may be the antithesis of what patients value or want.

## Data Availability

The total dataset that was examined during this study is not publicly available for confidentiality reasons. However, general information about it may be requested from the corresponding author, at mark.yaffe@mcgill.ca. All data generated or analyzed during this study are included in this published article.

## Abbreviations

CR: Charo Rodriguez
MJY: Mark J. Yaffe
RBH: Richard B. Hovey
